# Temporal Stability of Genetic Variability and Differentiation in the Three-Spined Stickleback (*Gasterosteus aculeatus*)

**DOI:** 10.1371/journal.pone.0123891

**Published:** 2015-04-08

**Authors:** Jacquelin DeFaveri, Juha Merilä

**Affiliations:** Ecological Genetics Research Unit, Department of Biosciences, University of Helsinki, Helsinki, Finland; University of Padova, ITALY

## Abstract

Temporal variation in allele frequencies, whether caused by deterministic or stochastic forces, can inform us about interesting demographic and evolutionary phenomena occurring in wild populations. In spite of the continued surge of interest in the genetics of three-spined stickleback (*Gasterosteus aculeatus*) populations, little attention has been paid towards the temporal stability of allele frequency distributions, and whether there are consistent differences in effective size (*N_e_*) of local populations. We investigated temporal stability of genetic variability and differentiation in 15 microsatellite loci within and among eight collection sites of varying habitat type, surveyed twice over a six-year time period. In addition, *N_e_*s were estimated with the expectation that they would be lowest in isolated ponds, intermediate in larger lakes and largest in open marine sites. In spite of the marked differences in genetic variability and differentiation among the study sites, the temporal differences in allele frequencies, as well as measures of genetic diversity and differentiation, were negligible. Accordingly, the *N_e_* estimates were temporally stable, but tended to be lower in ponds than in lake or marine habitats. Hence, we conclude that allele frequencies in putatively neutral markers in three-spined sticklebacks seem to be temporally stable – at least over periods of few generations – across a wide range of habitat types differing markedly in levels of genetic variability, effective population size and gene flow.

## Introduction

The study of evolution is ultimately about the study of changes in allele frequencies within populations over time. Allele frequencies in a given locus can change either due to deterministic (e.g. selection) or stochastic (e.g. migration, genetic drift, mutation) reasons [1]. In population genetics, as in evolutionary biology in general, allele frequency changes are more frequently and widely studied with synchronic (i.e. study of spatial genetic variation) than with allochronic approaches (i.e. study of temporal variation). With the notable exception of experimental evolution approaches undertaken in laboratory or mesocosms [2,3], the utility of allochronic approaches in population genetic studies of wild populations has traditionally been limited by access to historical samples [4,5]. Therefore, synchronic approaches have remained by far the most common way of inferring evolutionary transitions. However, inferences from patterns of spatial genetic variation at a given time point are subject to errors and biases, such as noise from intralocus sampling error [6], non-random sampling of relatives [6,7] or differentiation among sampled age classes [8]. Therefore, repeated sampling of the same localities at different time points provides an effective way of assessing if spatial genetic patterns—whether caused by neutral or selective processes—persist over time, and hence, can be reliably inferred from samples collected at one particular time point.

During the past decade, an increasing number of studies have investigated temporal changes in allele frequencies and population genetic parameters in the wild, both over long (i.e. many generations; [9,10]) and short (i.e. consecutive years [11,12]) time periods. Some have explored temporal stability of allele frequencies within high gene flow environments in order to assess the biological significance of low but statistically significant population differentiation (e.g. [11,13–16]). Others have been interested in temporal allelic shifts in small, closed populations [17], since stochastic effects such as drift are more likely to have a large effect on temporal genetic differentiation when effective population size is small [18]. However, simultaneous exploration of temporal stability of genetic parameters in populations with contrasting demographics/population structure (i.e. both with and without gene flow) is often not possible (but see [19] and [20] for interspecific comparisons) because many species do not demonstrate such contrasts in their population structure and demography, even over large areas (e.g. [16]).

In addition to informing us about population structure, temporal changes in allele frequencies in neutral loci can also allow for the estimation of effective population sizes (*N*_*e*_; reviews in: [18,21,22]). *N*_*e*_-estimates in turn provide important information that can be applied in management and conservation of wild populations [22,23]: Low *N*_*e*_ increases the loss of genetic diversity and can thereby impede populations’ capacity for adaptive change. Not surprisingly, temporal studies of genetic variability are particularly common in species of economic interest such as salmonids and marine fishes (reviews in [18,22,24,25]). However, species of more academic interest have less frequently been subject to rigorous tests of temporal stability of population genetic parameters.

The three-spined stickleback (*Gasterosteus aculeatus*) provides a case in point. This species has been subject to an increasing number of population genetic studies during the past decade (e.g. [26–33]). However, as far as we are aware, none of these studies have explicitly investigated the temporal stability of genetic variability and structuring of populations. For example, McCairns and Bernatchez [30] and Araguas et al. [34] each pooled temporal replicates sampled one year apart after finding no genetic differences between sampling years, and DeFaveri et al. [32] mentioned in passing that there was no differentiation among four Baltic Sea sites sampled six years apart. Hence, to what degree the observed patterns of structuring and variability reflect spatial, as opposed to temporal variability stemming from various factors capable of reducing effective population size [6,25), remains largely unknown. Moreover, strong reductions in genetic diversity in freshwater as compared to marine populations of three-spined sticklebacks (e.g. [31,35–37]) suggest that reductions in effective population size—and thereby pronounced generation-to-generation fluctuations in allele frequencies—are more likely to occur in freshwater as compared to marine localities. Likewise, given that the low degree of population differentiation in neutral marker loci in marine fish populations [38,39]—including sticklebacks (e.g. [31,32,40])—has been identified as making their allele frequency estimates disproportionally prone to sampling errors [6], studies in temporal stability of genetic parameters in marine stickleback populations are also warranted.

The aim of this study was to investigate temporal stability of within-population genetic diversity and among-population genetic differentiation in three-spined sticklebacks. To this end, we recorded allele frequencies in 15 putatively neutral microsatellite loci in eight different sampling locations over a six-year period, corresponding to approximately two to three stickleback generations (cf. [41]). In order to probe whether temporal stability differs among habitat types—and therefore, between populations likely to differ in their effective sizes and the amount of gene flow between them—the sampling was conducted in two pond and two lake (i.e. closed populations) and four marine (i.e. open populations) sites. Furthermore, different single-sample and temporal methods were used to estimate effective population sizes in different sampling sites with the expectation that they would be smaller for finite freshwater habitats than for marine habitats.

## Material and Methods

### Ethics statement

The research described in this paper was conducted in strict accordance with the Finnish and Swedish legislation. Fishing rights in Finland belong to the landowner according to the Finnish Fishing Law (5§ 27.5.2011/600) and since the sampling occurred in government owned areas, the fish were collected under appropriate national fishing licenses (allowing capture and killing of the fish) possessed by persons involved in sampling. No ethical permission was required (verified from Animal Experiment Board in Finland) for the described scientific sampling according to the Finnish Animal Conservation Law (7§ 28.6.2013/498). The samples from Sweden in 2009 were collected in accordance with Swedish fishery regulation SFS 1994: 1716, Chapter 2 § 4 with permits from Länstyrelsen Västerbotten (no. 620-4696-2009) and Länstyrelsen Västra Götlands Län (no. 623-41555-2009). The 2003 samples were provided to us by the Swedish National Board of Fisheries under their own permits. The fish were sacrificed by an overdose of MS-222 (tricaine methanesulfonate) immediately upon their capture. Hence, suffering before anesthesia was minimal.

### Sampling

**Fig 1:**
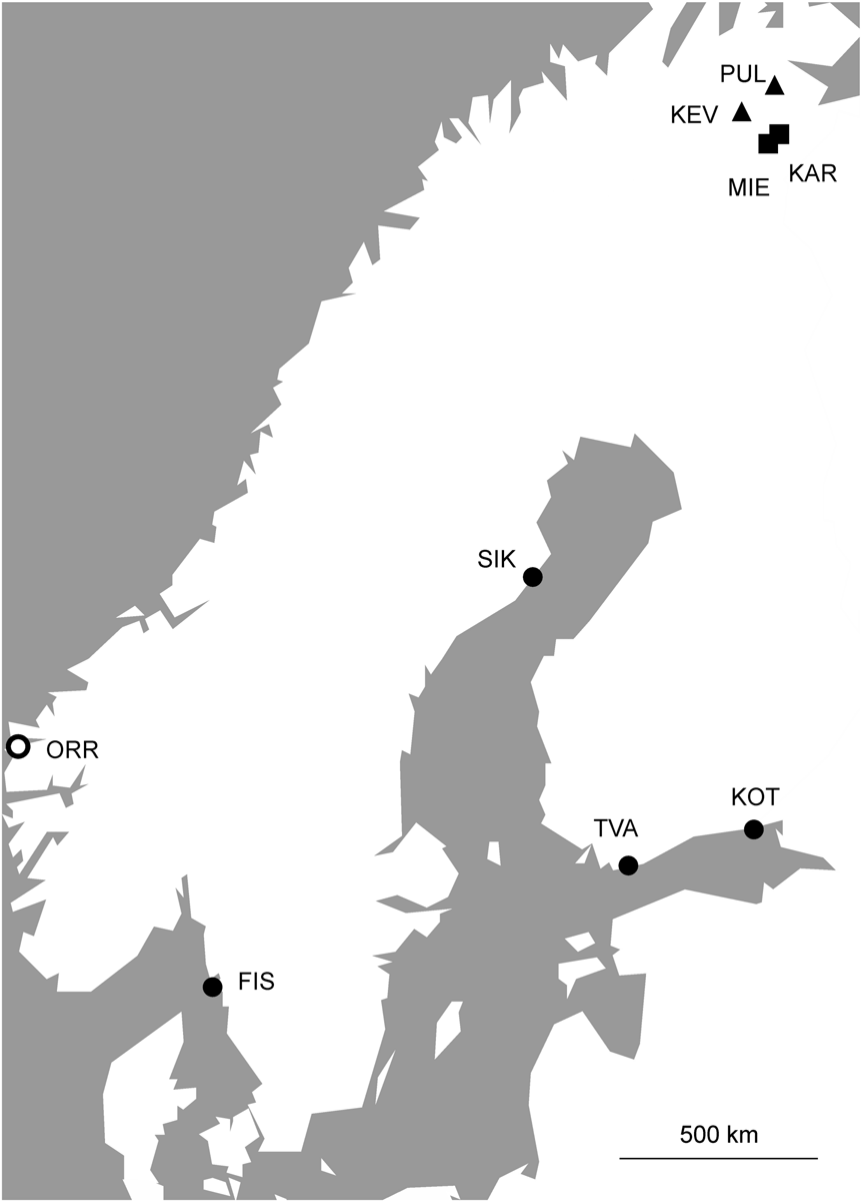
A map showing the geographic location of the eight sampling locations. Circles denote sea; triangles, lake; squares, pond populations. ORR indicates a source population to infer migration patterns and *Ne* in FIS according to the MLNE method (see text for details).

Adult sticklebacks were collected during the breeding season (May–June) in 2003 (n = 322) and 2009 (n = 327) from eight different localities (Table 1; Fig 1). Four of the localities are marine/brackish sites: one situated in the North Sea, and the others in the Bay of Bothnia and Gulf of Finland in the Baltic Sea. The data on 2009 samples from these four populations have earlier been used in ref [32]. The remaining four localities are freshwater sites from northern Finland, two of which are small ponds (approximately 100 m^2^; Table 1; Fig 1) and two are large lakes (1–12 km^2^; Table 1; Fig 1). The two pond populations are isolated, and hence, totally closed from migration. Although the two lake populations are connected to the Barent’s Sea by rivers, they are phenotypically [42] and genotypically [43] divergent from potential source populations, and hence there is no reason to expect on-going gene flow. After collection, the fish were preserved in ethanol.

**Table 1:** Basic information and summary statistics by sampling locality for the15 microsatellite loci.

	Locality	Coordinates	Area (km^2^)	Year	N	Age	H_E_	A	A_R_	A_P_	F_IS_	F_ST_
Baltic Sea	Fiskebäckskil (FIS)	58°15’05”N, 11°27’06”E	∞	2003	35 (30)	2.7 (2–4)	0.78	10.7	9.4	0.3	-0.02	0.000
		58°14’05”N, 11°24’06”E		2009	48 (42)		0.76	11.0	9.1	0.4	0.05	
Baltic Sea	Kotka (KOT)	60°27’18”N, 26°55’22”E	∞	2003	48 (45)	3 (2–4)	0.74	10.9	8.7	0.2	0.00	0.001
		60°33’55”N, 27°12’22”E		2009	43 (39)		0.75	11.1	9.0	0.3	0.02	
Baltic Sea	Tvärminne (TVÄ)	59°50’20”N, 23°12’15”E	∞	2003	32 (26)	3.6 (3–5)	0.75	9.5	8.8	0.3	0.02	0.000
		59°50’20”N, 23°12’15”E		2009	29 (28)		0.74	9.3	8.5	0.2	0.01	
Baltic Sea	Sikeå (SIK)	63°59’01”N, 20°53’17”E	∞	2003	32 (27)		0.75	9.8	8.6	0.1	-0.01	0.000
		64°09’34”N, 20°58’37”E		2009	35 (34)		0.74	9.7	8.6	0.2	0.02	
Lake	Kevojärvi (KEV)	69°45’01”N, 27°00’55”E	1.02	2003	46 (40)	3.7 (2–6)	0.56	6.3	5.1	0.1	-0.02	0.000
		69°45’01”N, 27°00’55”E		2009	32 (29)		0.52	5.6	5.0	0.1	0.01	
Lake	Pulmankijärvi (PUL)	69°58’46”N, 27°58’55”E	12.18	2003	42 (35)	2.8 (2–5)	0.63	7.0	6.0	0.1	-0.01	0.002
		69°58’46”N, 27°58’55”E		2009	44 (40)		0.62	6.5	5.5	0.0	0.05	
Pond	Mieraslompolo (MIE)	69°34’10”N, 27°14’41”E	0.08	2003	41 (34)	2.5 (2–5)	0.20	2.9	2.3	0.1	-0.07	0.000
		69°34’10”N, 27°14’41”E		2009	48 (47)		0.18	2.7	2.2	0.0	0.11	
Pond	Karilampi (KAR)	69°33’59”N, 27°14’35”E	0.13	2003	46 (45)	1.8 (1–3)	0.26	3.6	2.8	0.2	-0.01	0.004
		69°33’59”N, 27°14’35”E		2009	48 (44)		0.25	3.1	2.4	0.2	0.02	

N = sample size (number of complete genotypes scored), Age = average age in years (min—max) as determined in [42], H_E_ = expected heterozygosity, A = number of alleles, A_R_ = allelic richness, A_P_ = number of private alleles, F_IS_ = fixation index, F_ST_ = pairwise genetic distance [50] between samples collected from the same locality

### DNA extraction and genotyping

DNA extractions were performed from pectoral fin clips using a 10% Chelex -100 resin (Bio-Rad Laboratories, Richmond CA). Fifteen microsatellite loci (STN3, STN23, STN24, STN30, STN38, STN42, STN79, STN110, STN123, STN132, STN146, STN168, STN174, STN185, STN195, [32]) were amplified in 10 μl multiplex reactions with the following concentrations: 1 × Qiagen multiplex PCR master mix (Qiagen Inc. Valencia, CA, USA), 0.5 × Q-solution, 2 pmol of each forward (fluorescently labeled) and reverse primer and approximately 10–20 ng of template DNA. The PCR cycling profile was as follows: activation step at 95°C for 15 min, 30 cycles of denaturation at 94°C for 30 s, annealing at 55°C for 90 s, and extension at 72°C for 60 s. A final extension at 60°C for 5 min was included to complete the cycle. All PCR products were resolved with a MegaBACE 1000 automated sequencer (Amersham Biosciences) following 1:500 dilution, and alleles were scored with Fragment Profiler 1.2 software (Amersham Biosciences). Automatic binning was first used to designate allele sizes, and then sizes were edited by eye (JD). All genotype data used in this paper have been uploaded to Dryad repository: http://doi.org/10.5061/dryad.p15h2.

### Data quality

The program MICROCHECKER [44] was used to check for the presence of null alleles. Tests for deviations from Hardy-Weinberg equilibrium, as well as the extent of linkage disequilibrium between all pairs of loci in each sample, were conducted with the program FSTAT 2.9.3.2 [45]. Selective neutrality was tested for using two outlier detection methods: the coalescent-based FDIST method as implemented in LOSITAN [46], and the Bayesian method of Foll and Gaggiotti [47] as implemented in BAYESCAN. Since divergence can accumulate between habitats as a result of ecological differences [31,37] both tests were performed in a habitat-specific fashion (i.e. among lake, pond and sea populations separately).

### Estimates of genetic variability and differentiation

The expected heterozygosity (H_E_), number of alleles (A), allelic richness (A_R_), and inbreeding coefficient (F_IS_) were calculated for each population at each sampling point using FSTAT 2.9.3.2 [45]. The program HP-RARE 1.0 [48] was used to calculate private allelic richness (A_P_). Non-parametric tests were used to test for differences in diversity estimates between sampling years. Differences in allele frequency distribution were tested for with the genic differentiation option in the online program GENEPOP 1.2 [49], using Fisher’s exact probability tests. The degree of differentiation—both globally over all populations, as well as pairwise comparisons among populations—was estimated using Weir and Cockerham’s theta [50] following 1000 permutations in FSTAT 2.9.3.2 [45]. POPULATIONS 1.2 [51] software was used to construct a neighbor-joining tree based on pairwise chord distance (*D*_CE_; [52]) between populations. A Bayesian clustering algorithm was used to determine population structure within freshwater and marine habitats, as performed with the program STRUCTURE 2.3.3 [53]. The eight samples from the two pond and two lake populations were run together under the admixture model for five iterations for each value of K ranging from one to eight. Each run started with 50 000 burn-in steps followed by 100 000 MCMC repetitions. All other parameters were set to the default values. The eight samples from the four marine populations from the Baltic Sea were run under similar conditions, except sampling location was used as a prior in order to assist in detecting structure when levels of divergence are low [54]. The Structure Harvester web service [55] was used to determine and visualize the most probable value of K.

### N_e_ estimates

Given the challenges and uncertainties associated with estimation of effective population size (*N*_*e*_), we used six different methods—three temporal and three single-sample—to estimate *N*_*e*_ in each of the study sites. The rationale behind the use of all these methods was to affirm the robustness and reliability of the estimates by comparison: if multiple methods give similar estimates, more confidence can be placed on the conclusions (cf. [56]). We wish to further emphasize the fact that the primary goal in these comparisons was not to compare the performance of the estimators as such. Rather, the goal was to gain confidence in inferring possible *N*_*e*_ differences among the three different habitat types with the *a priori* expectation of *N*_*e* [Sea]_ > *N*_*e* [Lake]_ > *N*_*e* [pond]._

For the single-sample estimators, three methods were used to estimate inbreeding *N*_*e*_ from the multilocus genotype data of each sampling location at each time point.

The first was the linkage disequilibrium (LD; [57]) method, which uses the unbiased estimator of Burrow’s Δ [58] to test for non-random associations between unlinked loci. The random mating model was used for the LD option as implemented in the software N_E_ESTIMATOR v2 [59], which corrects for sample size bias and accounts for missing data [60]. Allele frequencies of less than 0.05 were excluded, and confidence limits were calculated by jackknifing over loci. The second was the sibship assignment method (SA; [61]), which uses sibship frequencies estimated from randomly sampled pairs of individuals as being sibs sharing one or two parents. The program Colony2 [61] was run under the full likelihood model with high precision, polygamous breeding systems for both sexes, and no prior information on candidate parents or sibship sizes. The third was the Bayesian method as implemented with the online program ONeSAMP [62], which uses approximate Bayesian computation (ABC) to calculate eight summary statistics—including LD—to estimate *N*_*e*_. Prior *N*_*e*_ estimates (lower and upper) were set to two and 500 for the freshwater populations, and two and 5,000 for the Baltic Sea populations.

For the temporal estimators, three methods were used to estimate the harmonic mean of variance *N*_*e*_ based on the samples taken at both time points for each sampling location.

The first was the moment-based method of Waples [63], which uses F statistics to calculate the standardized variance in allele frequency between sampling events. Three different options were used to measure *N*_*e*_: F_c_ [64], F_k_ [65], and the unbiased estimator F_s_ [66]. In the case of these F statistics, the *N*_*e*_ estimates were divided by the number of generations represented in the sample. The information on approximate generation length was obtained for all freshwater populations collected in 2003, as well as three Baltic Sea sites (FIS, KOT, TVA) collected in 2003, from the average age of breeding adults in the given population ([67]; see [41]). Since the average age in most populations was close to three (Table 1; [41]), this corresponded to a maximum of two generations. In the case of KAR, the average age was two, corresponding to three generations. N_E_ESTIMATOR v2 [59] was used to generate each estimate after excluding allele frequencies of less than 0.05, and confidence limits were calculated by jackknifing over loci. The second moment-based method was the pseudo-maximum likelihood method of Wang and Whitlock ([68]; MLNE), which relaxes the assumption of closed populations. As such, *N*_*e*_ and migration, *m*, were estimated jointly for the Baltic Sea populations since there is a high degree of gene flow in the Baltic Sea [28,32]. In these analyses, FIS was used as a source population for KOT, SIK and TVA, as earlier analyses of gene flow indicate that migration rate from North Sea to Baltic Sea is higher than the reverse [32]. An Atlantic population from the coast of Norway (Orrevannet; 58°44’N 05°31E, see [28]; Fig 1) was used as a source population for FIS. Since the freshwater populations are isolated and therefore are not likely experiencing gene flow with other populations, only *N*_*e*_ was estimated. Two generations were assumed for all populations except KAR, in which three generations were assumed. The upper *N*_*e*_ priors were set to 500 for the freshwater populations and 5,000 for the Baltic Sea populations. The third method was the likelihood-based method of Berthier et al. ([69]; TM3), which uses coalescence theory and Bayesian prior information to estimate *N*_*e*_. The same upper *N*_*e*_ priors were used as in the ABC and MLNE methods.

## Results

### Data quality

Although there was evidence of null alleles and Hardy-Weinberg deviations at some loci in some populations, there were no consistent patterns across any locus, populations or habitats. Similarly, there was no consistent pattern of linkage disequilibrium in any pair of loci across all populations. Neither of the outlier tests detected divergent loci in the pond and lake populations, however one locus (STN146) was detected as an outlier with both methods in the Baltic Sea populations. Although visual inspection of the data revealed that the elevated divergence observed in this locus arose from spatial, not temporal, shifts in allele frequencies, it was nevertheless eliminated from further analyses.

### Genetic variability

**Fig 2:**
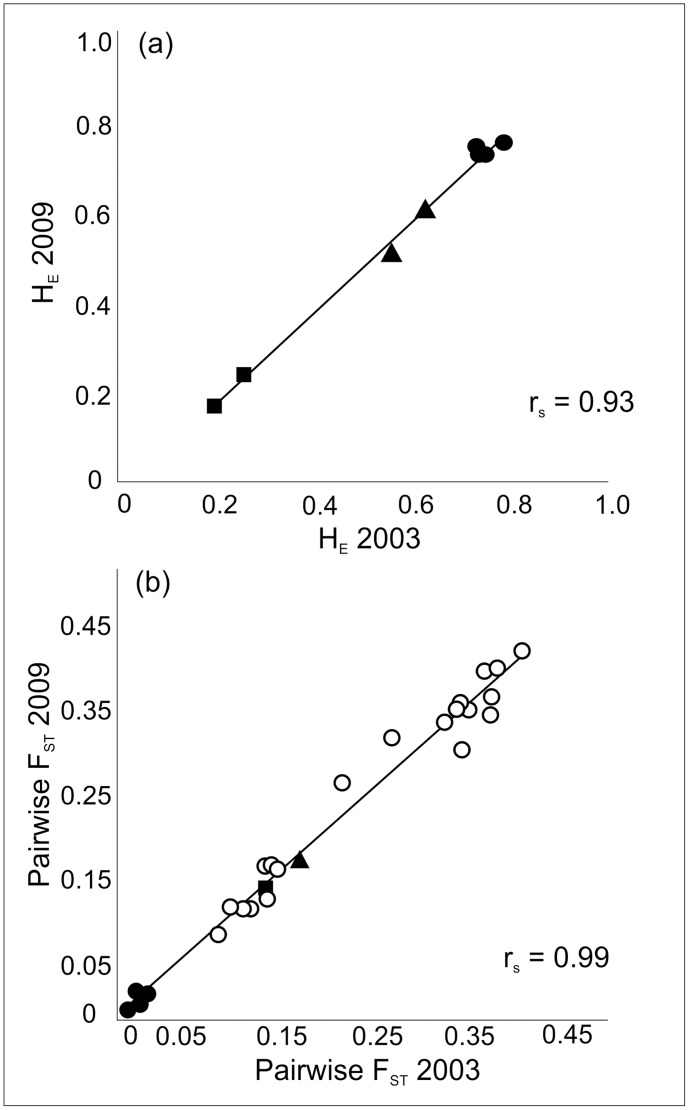
Correlation between (a) expected heterozygosity and (b) pairwise *F*_ST_ values from two different sampling occasions. Closed circles denote pairwise *F*_ST_ among sea populations; triangles among lake populations; squares among pond populations; open circles between habitat comparisons.

There was significant genetic heterogeneity among populations in all genetic diversity measures: marine populations were genetically more variable than the lake populations, which were more variable than the pond populations (Table 1). However, there were no temporal differences in the allele frequencies in any loci in any of the populations between the two sampling occasions (Fisher’s exact probability tests, P > 0.05). Accordingly, different estimates of genetic variability were similar (Kruskal-Wallis test, P > 0.05 for H_E_, A, and A_R_) and strongly correlated (*r*_*s*_ = 0.77–0.93; n = 8, P ≤ 0.001) between the two sampling occasions (e.g. Fig 2a). F_IS_ values were small and similar in all populations and across the two sampling periods (Table 1).

### Genetic differentiation

**Fig 3:**
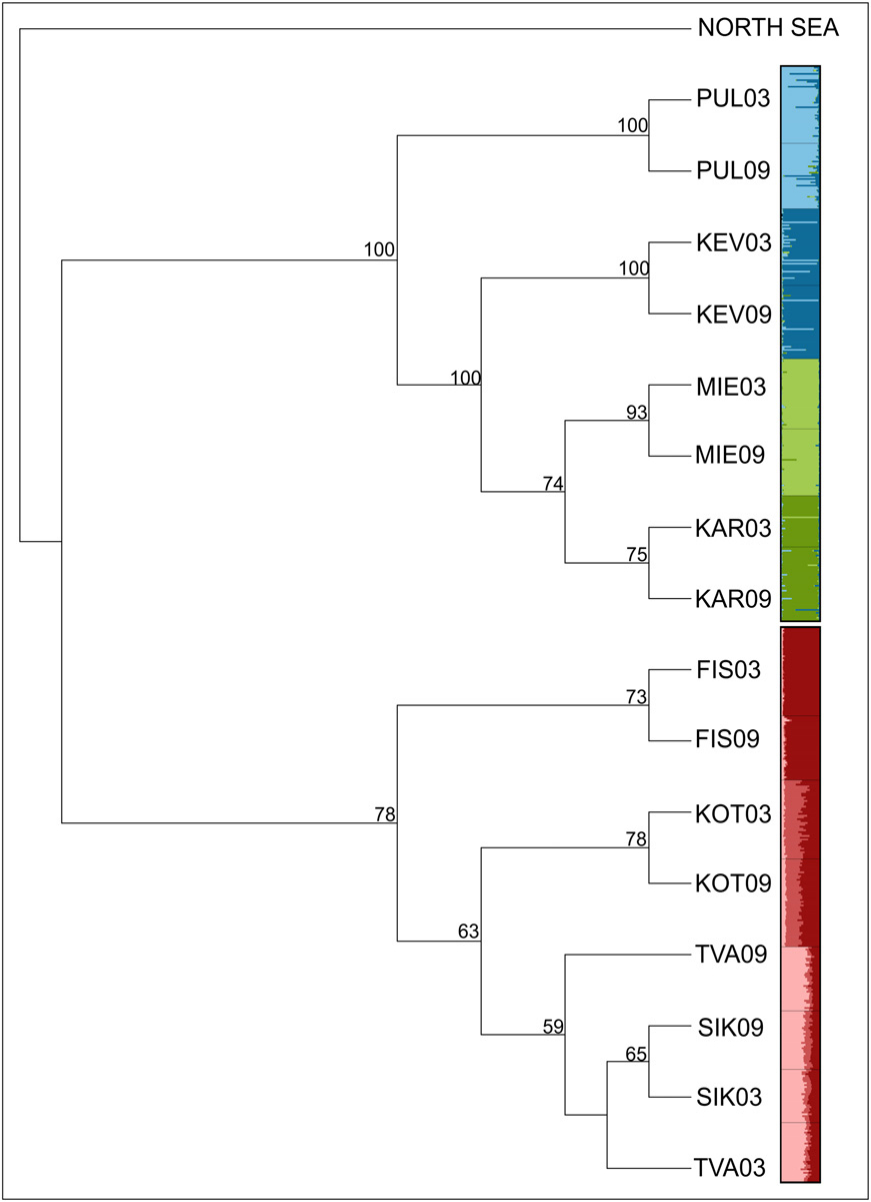
Neighbor-joining tree based on *D*_CE_ distances [52], rooted with a population from the North Sea. Bootstrap values above 50% are indicated at nodes. Genetic clusters as detected with STRUCTURE [53] are indicated at branch tips.

The overall *F*_ST_ across all populations was 0.199 (95% CI: 0.156–0.238, P < 0.05) in 2003 and 0.209 (95% CI: 0.107–0.246, P < 0.05) in 2009, and the pairwise estimates across populations over time were strongly positively correlated (*r* = 0.99; n = 8, P < 0.001; Fig 2b). Within each habitat type, overall *F*_ST_ was similar between years (Table 2). Accordingly, the temporal replicates branched together in the neighbor-joining tree, with high bootstrap support for the freshwater populations (Fig 3). STRUCTURE also assigned temporal replicates to the same genetic cluster for each population (Fig 3). Each of the freshwater sampling locations were assigned as independent clusters (K = 4), and the most likely K for the sea populations was three (Fig 3). Among all populations, 20.36% of the genetic variation was attributed to differences among habitats, but the variance component due to temporal changes within localities was negative and non-significant (Table 3). This was true whether all populations were analyzed together or when each habitat was analyzed separately (Table 3).

**Table 2:** Comparison of estimates of population differentiation (*F*_ST_) between sampling years (2003 and 2009) in different habitat types.

Habitat	2003	95% CI	2009	95% CI
Baltic Sea	0.004	0.002–0.007	0.009	0.002–0.015
Lake	0.174	0.115–0.235	0.174	0.123–0.222
Pond	0.141	0.023–0.272	0.148	0.038–0.270
Overall	0.199	0.156–0.238	0.209	0.107–0.246

**Table 3:** Hierarchical partitioning of spatial and temporal variation in allele frequency data with AMOVA.

Source of variation	Degrees of freedom	Sum of Squares	Variance component	% of variation	p
Among habitat type	2	962.56	1.103	20.38	<0.001
Among years	1	5.55	-0.132	-2.64	0.986
Baltic Sea	1	5.08	-0.018	-0.33	0.912
Lake	1	6.76	-0.445	-9.39	0.328
Pond	1	1.99	-0.136	-7.58	0.322

### *N*_*e*_ estimates: Single-sample methods

**Fig 4:**
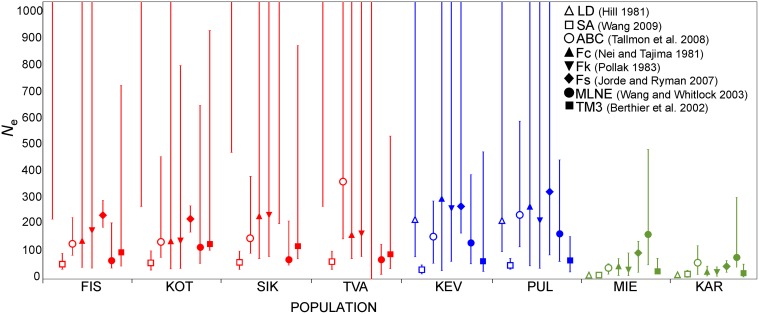
Estimates of effective population size in each of the sea (red), lake (blue) and pond (green) populations. Infinite confidence intervals are indicated with lines without caps. Single-sample estimates (LD, SA, ABC) represent harmonic mean of the two sampling periods. MLNE estimates for sea populations are assuming migration whereas those for the freshwater populations assume no migration.

The LD method yielded infinite *N*_*e*_ estimates for all the sea populations (Fig 4; S1 Table). The *N*_*e*_ estimates were an order of magnitude higher for the lake populations (average = 238) as compared to the pond populations (average = 13; Fig 4). The SA method yielded the lowest *N*_*e*_ estimates across all populations, with the narrowest confidence intervals (Fig 4; S1 Table). The sea populations had similar *N*_*e*_ estimates (average = 53) to the lake populations (average = 43), while the pond populations had the lowest (average = 15; Fig 4; S1 Table). The ABC method also yielded *N*_*e*_ estimates that were similar across sea (average = 147) and lake (average = 213) populations. As with the other methods, *N*_*e*_ estimates in the pond populations were significantly smaller (average = 54) and more precise (narrower confidence intervals; Fig 4; S1 Table).

### *N*_*e*_ estimates: Temporal methods

The standard moment-based method of Waples (1989) yielded similar results whether calculated according to Nei and Tajima (F_c_: [64]), Pollak (F_k_: [65]) or Jorde and Ryman (F_s_: [66]; Fig 4; S1 Table). *N*_*e*_ estimates were slightly but not significantly higher in the lake (average = 271) than sea (average = 163) populations. Most estimates yielded infinite confidence intervals, except the F_s_ method for some of the sea populations (Fig 4; S1 Table). *N*_*e*_ estimates for the pond populations were an order of magnitude lower (average = 38) than for the lake and sea populations, with narrower confidence intervals (Fig 4; S1 Table). When calculated with the assumption of migration, MLNE yielded the lowest *N*_*e*_ estimates among the sea populations (average = 78; Fig 4; S1 Table). Assuming no migration, *N*_*e*_ was similar across all freshwater populations, and was slightly higher than in the sea populations (average = 135; Fig 4; S1 Table). The *N*_*e*_ estimates from TM3 yielded similar patterns as the single-sample SA method, where *N*_*e*_ was highest in the sea populations (average = 110), lowest in the pond populations (average = 16) and intermediate in the lake populations (average = 60; Fig 4; S1 Table).

## Discussion

The most salient finding of this study was that, despite the differing levels of genetic diversity, gene flow and habitat size, allele frequencies as well as genetic variability and differentiation measures within and among three-spined stickleback populations from both marine and freshwater habitats were temporally stable over the six year time period. This suggests that, in spite of the potential for sampling artifacts and low signal-to-noise ratio to affect estimated genetic parameters, these issues were of negligible concern. It is particularly noteworthy that this was true in the case of small isolated populations as well as in larger interconnected populations. Likewise, using different methods to estimate effective size of stickleback populations from different habitats, we found fairly consistent support for relatively small effective sizes (tens to hundreds) in all habitat types, although the census population sizes in this species are likely to be orders of magnitudes higher (e.g. [70–72]). In the following, we discuss these findings and their implications to studies of genetic population structure, and that of the three-spined stickleback in particular.

### Temporal stability of allele frequencies

Most population genetic studies employ allochronic approaches to assess shifts in allele frequencies, with the underlying assumption of temporal genetic stability. Our results demonstrating short-term stability of genetic parameters in multiple three-spined stickleback populations across a range of habitat types validate this assumption for this species. This finding may not be surprising considering the relatively short time period—roughly two to three stickleback generations—between sampling events. The probability of detecting significant allele frequency differences when samples are collected few years apart may be low (e.g. [73]). Moreover, the temporal stability in genetic parameters in the high gene flow Baltic Sea environment was expected (see: [9,11,16,74–77] for similar examples), since genetic diversity is likely to be maintained by migration [78,79]. However, the stability in the two small and isolated pond populations is noteworthy because the stochastic component to variance in allele frequencies in small populations is expected to be large [80,81].

### Temporal stability in genetic diversity

Genetic drift is typically stronger in small than in large populations. Specifically, the rate of loss of diversity is inversely proportional to the effective population size (i.e. 1/2*N*_*e*_; [80]). Therefore, loss of diversity via drift can be expected to be faster—particularly in the absence of migration—in small than in large populations. Moreover, inbreeding and the accumulation of deleterious mutations can accelerate loss of genetic variation in small populations [18,78]. In accordance with these expectations, we found that the genetic diversity in the freshwater populations was substantially lower than that in the marine populations (see also: [28,35,36] for similar evidence from this species), and yet, genetic diversity measures remained stable over the study period in all sampling sites. This temporal stability of genetic parameters in the small pond populations being similar to those of the larger lake and sea populations could come about if the difference in *N*_*e*_ between the pond and other populations was too small to generate noticeable allele frequency shifts over the short time span encompassed in our study (cf. [82]). For example, assuming *N*_*e*_ of 30 in the pond populations and 150 in the sea populations, the rate of loss in the former would “only” be five times faster than in the latter. Hence, as evidenced by their low genetic diversity, the pond populations are likely more susceptible to the negative consequences of drift than the larger lake and Baltic Sea populations. In fact, most point estimates of *N*_*e*_. for pond populations were lower than 50, which is far below the minimum short-term *N*_*e*_ required to the ensure potential for long-term persistence in the face environmental changes and to avoid inbreeding problems [78,83,84].

### Temporal stability in population structure

Demonstrating temporal stability in the patterns and degree of population structure is a powerful way of confirming the reliability of observed spatial genetic patterns. For example, Tessier and Bernatchez [85] sampled salmon populations over several generations to validate their earlier finding of surprisingly high divergence within a single river system [86]. Temporal sampling is especially relevant in the case of subpopulations interconnected by gene flow, such as those of marine fishes in which low but statistically significant levels of differentiation are often observed [38]. Several studies have utilized temporal replicates—spanning few to many generations—to infer the biological significance of weak differentiation (e.g. [9,11,13]), or to clarify contrasting patterns of differentiation [74]. Moreover, when sample sizes are small in comparison to population size, as is likely the case with many marine fishes, accuracy of allele frequency estimates is reduced by small sample sizes [60,63]. Hence, our finding of stability in genetic composition among the Baltic Sea samples suggests that sample size and sampling errors did not influence the observed patterns. In addition, this result indicates that the barriers to gene flow between the North Sea and Baltic Sea have remained stable over several years. Similar findings have been reported in other fish studies comparing temporal stability genetic parameters across the Baltic—North Sea transition [14,16,87].

Although differential allele frequency changes are expected in populations subject to different levels of gene flow [88], the small, isolated pond populations showed the same degree of temporal stability in patterns and degree of population differentiation as the large, interconnected Baltic Sea populations. Albeit only one year had elapsed between sampling periods, Araguas et al. [34] also failed to find significant temporal heterogeneity in differentiation between endangered, isolated freshwater stickleback populations in the Iberian Peninsula. Similarly, Raeymaekers et al. [89] reported weak but significant temporal differentiation among fresh- and brackish water stickleback populations—connected by gene flow—collected several times during two years. McCairns and Bernatchez [30] also noted strong clustering of temporal samples collected from the high gene flow St. Lawrence estuary. With six years elapsed between sampling events, our current study represents—to the best of our knowledge—the longest time period over which temporal stability of allele frequencies in presumably neutral marker genes in stickleback populations has been assessed. Nevertheless, our results accord with those of earlier studies in which both isolated and interconnected populations appear to be stable over short term. It is important to note that despite the apparent stability in neutral population structure of sticklebacks, several studies have identified significant seasonal and temporal changes in allele frequencies of the locus underlying lateral plate number differentiation [89–91]. Similar findings were also reported by Fraser et al. [19], who noted that although temporal replicates of guppy populations were stable in neutral loci, there were significant changes in the frequency of different MHC alleles. Hence, the stability of allele frequencies in neutral loci may differ from those subject to natural selection—a situation analogous to the differential degree of spatial genetic differentiation in neutral and selected loci (e.g. [92,93]).

Finally, we note that the results of this study in respect to the degree and patterns of genetic differentiation among Baltic and North Sea populations are largely in agreement with earlier studies conducted across these sites [28,32,36,94]. In general, as shown also by our results, the degree of neutral genetic differentiation is usually clearest between the North Sea and Baltic Sea sites, while little differentiation is usually detected among the inner Baltic Sea sites (see also [36]). However, several markers located within or close to genes of functional importance display marked differentiation even within the inner Baltic Sea [32]. The shallow genetic structuring in neutral genes within the Baltic Sea is also likely to explain why the patterns of genetic differentiation recovered in the present and earlier studies [28,94] are not always exactly concurrent. Part of these discrepancies may occur because the markers used in the present study were not exactly the same as those in the previous study [32,94], and/or that the sample size in this study were higher than in earlier studies [94]. For instance, the degree of genetic differentiation (as reflected in F_ST_) among the marine sites SIK and KOT, as well as between KOT and TVA, were significant in this but not in a previous study [94]. Yet, this discrepancy occurred only in two of the six possible comparisons and concurrence between the F_ST_-estimates across the two studies is high (r_s_ = 0.84, P = 0.036).

### Effective population size

Although the assumptions associated with estimating effective population size are likely violated in many studies [18], both temporal and single-sample methods have remained popular tools in population and conservation genetics. While many studies have reported some degree of congruence among estimates obtained with different methods (e.g. [56,95, 96], a high degree of variation surrounding estimates is more of a rule, rather than exception. This applies to some degree to our study as well. Like most other studies, we found that *N*_*e*_ estimates were most precise when small. This was most often seen with all methods in the pond populations, and with the single-sample sibship analysis method in all populations. However, there is likely little probability of sampling sibs in our scheme, since sibs are unlikely to be present among breeders [97], at least not in the samples from the sea. In fact, the sibship method may be biased for large *N*_*e*_ [61], and hence not be very appropriate for the sea populations used in our study. Therefore, although precise, the sibship results may not be the most appropriate given our sampling design. In general, the confidence intervals for *N*_*e*_ estimates in the sea and lake populations obtained with most other methods included infinity, which is likely to be a reflection of weak genetic signal relative to sampling noise: the precision of *N*_*e*_ estimates declines as *N*_*e*_ increases [63]. However, it is noteworthy that irrespective of the method used, the lower confidence limits were consistently (ignoring the LD-method estimates; see Fig 4) higher for sea and lake as compared to pond populations.

In general, the point estimates of *N*_*e*_ of the sea populations were lower than expected, as those of many marine fishes are at least an order of magnitude higher (e.g. [16,75,98]). This could derive from the fact that most methods used here assume population isolation and that shifts in allele frequencies occur strictly as a result of drift without introduction of new alleles from migrants [8,66,68]. However, gene flow may inflate diversity and variance in allele frequencies, creating a signal of instability and therefore yield low *N*_*e*_ estimates (e.g. [15,68]). Likewise, episodic pulses of gene flow from divergent populations—such as large influxes of freshwater or anadromous sticklebacks to a given sea area—could explain the low *N*_*e*_ in marine sites [99]. We tried to avoid this potential problem firstly by performing clustering analysis and subsequently estimating *N*_*e*_ according to the identified genetic clusters, rather than on the basis of predefined populations based on sampling location. Secondly, we used the method of Wang and Whitlock [68] which accounts for gene flow from a predefined source population. Interestingly, this method yielded slightly lower *N*_*e*_ estimates than the methods assuming no migration—a finding encountered in several other studies too (e.g. [10,56,95,100,101]). In addition, violation of the assumption of discreet generations can lead to downward bias in *N*_*e*_ estimates [8,66], but corrections for overlapping generation require considerable life-history information as well samples from consecutive cohorts (e.g. [102]) which are not available for our study.

The low *N*_*e*_ estimates for sea and lake populations of three-spined stickleback populations are puzzling given that census population sizes (*N*_*c*_), even in small ponds, can be in the range of thousands (e.g. [72]), and those in the Baltic Sea most likely in the millions (e.g. [70,71]). Hence, a conservative assumption of *N*_*c*_ = 10 000 for freshwater populations and *N*_*c*_ = 1 million for sea populations would yield in *N*_*e*_/*N*_*c*_ ratios far below the usual ≈10% observed in studies of wild animal populations [103]. Although other studies of marine species have reported similar findings [18,22], we also observed this in the lake populations. As discussed above, violation of assumptions (e.g. no migration, discreet generations) together with low signal-to-noise ratio may explain the low point estimates of *N*_*e*_ in the lake and sea samples: the genetic signal may simply be too weak to discern between *N*_*e*_ of moderate and large size (cf. [6]). It is also worth pointing out that genotyping errors and non random sampling can inflate variance in allele frequencies, and thereby lead to downwardly bias *N*_*e*_ estimates. However, we have no reason to assume that these sources of error would differ among habitat types, and the genotyping error rates for the used loci are likely to be low as they were selected for this study on the basis of being easily scoreable. Likewise, while non random sampling of individuals is hard to discount without extensive resampling of individuals in a given locality, the high degree of temporal stability in all genetic parameters in this study speaks against such possibility.

As pointed out by Palstra and Ruzzante [18], the possibility of low signal-to-noise ratio could be investigated by substantially increasing sample sizes and thereby also the signal-to-noise ratio, although it is noteworthy that when we combined the two temporal samples in the single-sample methods, estimates remained unchanged (data not shown). Nevertheless, regardless of the absolute magnitude of *N*_*e*_-values, the sea and lake populations appear to have substantially higher (and likely underestimated) *N*_*e*_s than the pond populations: for most estimators, both the lower and upper confidence intervals, as well as the point estimates, were higher for sea and lake populations than for pond populations. In spite of the uncertainties involved with *N*_*e*_ estimates, the temporal changes in allele frequencies in all habitat types were small over the six year study interval, and hence, from the point of view estimation of genetic population structure, negligible.

### Conclusions

In conclusion, the results of this study demonstrate that genetic diversity and population structuring among three-spined stickleback populations are temporally stable over short term: this holds true for both small isolated freshwater populations as well as for large, interconnected marine populations. This result suggests that small scale temporal variation in sampling scheme is unlikely to bias or otherwise influence population genetic inference based on allele frequencies in neutral marker genes in this species. Although point estimates of effective population size varied depending on the estimation method, two fairly robust conclusions about them are possible. First, as expected, the effective sizes appear to be smaller in the pond than in lake and sea populations. Second, the census population sizes of sticklebacks are likely to be orders of magnitudes higher than the effective population sizes, at least in the isolated pond populations where the *N*_*e*_ estimates were most precise. Future studies utilizing more widely separated temporal cohort-based samples could be informative in gaining better estimates of effective size of contemporary populations (cf. [101]).

## Supporting Information

S1 Table Effective population size estimates for three-spined sticklebacks. (XLSX)
